# Medical condition and care of undocumented migrants in ambulatory clinics in Tel Aviv, Israel: assessing unmet needs

**DOI:** 10.1186/s12913-017-2421-y

**Published:** 2017-07-14

**Authors:** Zohar Mor, Yuval Raveh, Ido Lurie, Alex Leventhal, Roni Gamzu, Nadav Davidovitch, Orel Benari, Itamar Grotto

**Affiliations:** 1 Tel Aviv Department of Health Tel Aviv, 12 Ha’arba’a St, 6473912 Tel Aviv, Israel; 2 Faculty of Medicine, Tel Aviv University, Tel Aviv, Israel; 3 Faculty of Medicine, Hebrew University, Jerusalem, Israel; 4 Physician for Human Right (PHR-I), Tel Aviv, Israel; 5 Kfar Saba Adult Clinic, Shalvata Mental Health Center, Hod Hasharon, Israel; 6 Administration Department, Tel-Aviv Sourasky Medical Center, Tel Aviv, Israel; 7 Faculty of Health Sciences, Ben Gurion University of the Negev, Beer Sheva, Israel; 8 Lev el Lev Clinic, Ministry of Health, Tel Aviv, Israel; 9 Public Health Services, Ministry of Heath, Jerusalem, Israel

**Keywords:** Health inequalities, Health needs, Immigration, Israel, Medical insurance

## Abstract

**Background:**

Approximately 150,000 undocumented migrants (UM) who are medically uninsured reside in Israel, including ~50,000 originating from the horn of Africa (MHA). Free medical-care is provided by two walk-in clinics in Tel-Aviv. This study aims to compare the medical complaints of UM from different origins, define their community health needs and assess gaps between medical needs and available services.

**Methods:**

This cross-sectional study included a random sample of 610 UM aged 18–64 years, who were treated in these community clinics between 2008 and 2011. The study compared UM who had complex medical conditions which necessitated referral to more equipped medical settings with UM having mild/simple medical conditions, who were treated at the clinics.

**Results:**

MHA were younger, unemployed and more commonly males compared with UM originating from other countries. MHA also had longer referral-delays and visited the clinics less frequently.

UM with complex medical conditions were more commonly females, had chronic diseases and demonstrated longer referral-delays than those who had mild/simple medical conditions. The latter more commonly presented with complained of respiratory, muscular and skeletal discomfort. In multivariate analysis, the variables which predicted complex medical conditions included female gender, chronic illnes and self-referral to the clinics.

**Conclusions:**

The ambulatory clinics were capable of responding to mild/simple medical conditions. Yet, the health needs of women and migrants suffering from complex medical conditions and chronic diseases necessitated referrals to secondary/tertiary medical settings, while jeopardizing the continuity of care. The health gaps can be addressed by a more holistic social approach, which includes integration of UM in universal health insurance.

## Background

Israel has been confronted with a new phenomenon of becoming the destination of more than 50,000 migrants from the horn of Africa (MHA) since 2007, the majority (~75%) of whom originated in Eritrea [1]. It is estimated that an additional ~15,000 undocumented migrants (UM) from other countries and 90,000 non-citizens whose tourist visas have expired are currently staying in Israel [2]. Most of these UM are residing in Tel-Aviv, which is the economic capital of Israel, especially in its southern neighborhoods [3].

As the majority of these UM do not have a legal work permit be employed in Israel, they work unofficially, mostly in occupations of labor and services. As they lack a civil status, they are excluded from the National health insurance and other social benefits which are universally provided to Israeli citizens, and are therefore expected to pay from their own funds if they need medical care. During their stay in Israel, migrants may be exposed to several medical risks, such as physical injuries, adverse occupational exposures, acquiring or exacerbation of infectious diseases and other natural causes of morbidity.

In cases of urgent medical conditions, uninsured UM can attend emergency rooms in general hospitals. Ambulatory medical care is provided free of charge only by two community clinics situated in south Tel Aviv. One has been operated by the non-governmental organization “Physicians for Human Rights- Israel” (PHR-I) since 1998 [4], and the other by the Ministry of Health (MoH) since 2008. Medical services in these clinics include medical examinations mainly by general physicians and basic treatment [5]. These clinics also provide secondary medical care, but it is subject to the expertise of the physicians who volunteer at the clinics. In cases where the physician is unable to evaluate or treat the patient at the clinic, the patient is then referred for further laboratory testing, imaging or consultation by a medical expert, or to the emergency room in severe cases. Sexually transmitted infections [6] and tuberculosis [7] are managed by designated clinics, which are funded by the MoH.

Migrants’ use of medical service is unique, as they may have different health perceptions or beliefs and different health-seeking behavior than that of local citizens [8]. They may also not be aware that the ambulatory service is free and unrelated to their legal status in Israel. In addition, some do not live in Tel Aviv or are hesitant to use the services due to logistic, linguistic or cultural barriers [9, 10].

The purpose of the study was to compare the medical complaints of UM from different origins, define their community health needs and assess gaps between their medical needs and the available services in the community, and thereby to suggest possible improvements in medical care in the light of the current migration policy.

## Methods

This cross-sectional study included a random sample of 610 UM, aged 18–64 years, who were treated at the PHR-I or the MoH community clinics between 2008 and 2011. Patients who were Palestinians or tourists were excluded from the study, as they may have had alternative routes to seek medical care. Only the first visit of each study participant was recorded, and the random sample included the first 3 visits recorded for each month in both clinics. The data for this study were extracted from the medical files and included demographic characteristics, the patient’s major complaint, the body system which was involved, referral delay (time from the beginning of the symptoms to the first doctor’s visit) and the complexity of disease, which was classified as simple- if the medical condition was treated at the primary care level, moderate- if the primary physician asked for further laboratory tests, imaging or secondary care consultation, and complex- if the patient was referred to a secondary care setting or emergency room, as the medical condition could not be responded at the primary clinic.

MHA were compared with those originating from other countries. Additionally, UM who were referred from the walk-in clinics to another medical setting due to the limited capacity of the clinics were compared with those whose medical problem was treated fully at the premises of the clinics. Numerical data were compared by the chi-square and the Student’s *t*-tests for categorical and continuous variables, respectively. *P* values less than 5% were regarded as statistically significant. Variables which were statistically significant in the univariate analysis were included in the logistic regression to identify variables predicting complex medical conditions among UM.

This study was approved by the Institutional Review Board of the Wolfson Hospital, Holon, Israel (WOMC-11-084).

## Results

During the study period, 6820 and 4146 migrants visited the PHR-I and MoH clinics, respectively. The random sample captured 302 (4.4%) patients from PHR-I and 308 (7.4%) from the MoH clinics. MHA were more commonly males and younger than UM originating in other countries, their stay in Israel was relatively shorter and they were more commonly unemployed (Table 1). Additionally, MHA had longer referral delay and visited the clinics less frequently than UM originating in other countries.

**Table 1:** Comparison between undocumented migrants originating in the horn of Africa with migrants from other countries who visited ambulatory clinics in Tel Aviv, 2008–2011

Variable [vs. compared group]	Migrants from the horn of Africa *N* = 310 (%)	Migrants from other countries *N* = 300 (%)	*P*
MoH clinic [vs. PHR-I clinic]	159 (51.3)	149 (49.7)	0.7
Age: median	27 (IQR: 23–33)	34 (IQR:28–41)	*>0.001*
Female gender [vs. male]	67 (21.4)	127 (42.1)	*>0.001*
Length of stay in Israel [months]: median	9 (IQR: 4–16)	18 (IQR: 6–36)	*>0.001*
Married [vs. single]	112 (40.6)	99 (16.8)	0.4
Employed [vs. unemployed]	46 (71.9)	68 (86.1)	*0.04*
Disease [vs. injury]	277 (91.4)	267 (91.10)	1
Acute disease [vs. chronic]	199 (65.0)	211 (71.3)	0.1
Mild/simple symptoms [vs. moderate or complex]	193 (62.3)	170 (56.7)	0.2
Months from symptoms to Doctor’s visit: median	1.25 (IQR: 0.25–6)	0.7 IQR: (0.25–3)	*0.006*
Number of further visits to the clinic: median	1 (IQR: 1–2)	1 (IQR: 1–3)	*0.03*
Respiratory complaints	49 (16.6)	57 (19.5)	0.4
Muscular and skeletal complaints	56 (19.0)	64 (21.8)	0.4
Referral to other medical settings	113 (36.5)	124 (41.3)	0.2

*MoH* Ministry of Health

*PHR-I* Physicians of Human Rights

*IQR* Intra quadrantile range

UM who had complex medical conditions were more likely to visit the MoH clinic than the PHR-I clinic (Table 2). Those who had more complex medical condition were more commonly females, had chronic illnesses, demonstrated longer referral delay and their medical condition necessitated referral to better equipped medical settings. Interestingly, this referral delay did not achieve statistical significance once controlled for age and for the migrants’ length of stay in Israel (data not shown). Those who complained of mild/simple medical conditions more commonly had respiratory, muscular and skeletal discomfort. No significant statistical differences in complexity of disease were found between MHA and those originating from other countries. In the multivariate analysis, self-referral to the PHR-I clinic, female sex, and having chronic medical conditions predicted more complex medical conditions (Table 3).

**Table 2:** Comparison between undocumented migrants who had mild/simple medical conditions with those who had complex medical conditions who visited ambulatory clinics in Tel Aviv, 2008–2011

Variable [vs. compared group]	Mild/Simple *N* = 362 (%)	Moderate/Complex *N* = 247 (%)	*P*
MoH clinic [vs. PHR-I clinic]	220 (60.6)	88 (35.6)	*>0.001*
Age: median	30 (IQR: 25–37)	31 (IQR: 25–39)	0.2
Female gender [vs. male]	90 (25.1)	103 (41.2)	*>0.001*
Origin [horn of Africa vs. other countries]	193 (53.2)	170 (68.8)	0.2
Length of stay in Israel [months]: median	12 (IQR: 4.5–24)	12 (IQR: 5–30)	0.4
Married [vs. single]	127 (38.7)	84 (38.7)	1
Employed [vs. unemployed]	66 (82.5)	48 (76.2)	0.4
Disease [vs. injury]	324 (91.3)	220 (91.3)	1
Acute disease [vs. chronic]	264 (73.3)	146 (60.3)	*0.001*
Months from symptoms to Doctor’s visit: median	0.3 IQR: (0.2–3)	2 (IQR: 0.5–6)	*>0.001*
Number of further visits to the clinic: median	1 (IQR: 1–2)	1 (IQR: 1–3)	0.5
Respiratory complaints	77 (22.2)	29 (12.0)	*0.002*
Muscular and skeletal complaints	79 (22.8)	41 (17.0)	*0.01*
Referral to other medical settings	137 (37.7)	140 (56.7)	*0.001*

*MoH* Ministry of Health

*PHR-I* Physicians of Human Rights

*IQR* Intra quadrantile range

**Table 3:** Multivariate analysis identifying factors predicting moderate or complex medical conditions

Variable	Odds ratio (95% confidence interval)	*P*
PHR-I clinic	2.7 (1.8–4.0)	*<0.001*
Female gender	1.4 (1.2–1.8)	*0.005*
Months from symptoms to Doctor’s visit	1.0 (0.9–1.1)	0.6
Chronic disease	2.2 (1.5–3.1)	*<0.001*
Respiratory complaints	1.4 (0.9–2.4)	0.2

*MoH* Ministry of Health

*PHR-I* Physicians of Human Rights

## Discussion

This first study assessing the ambulatory health needs of UM demonstrated that those who presented with more complex medical conditions were more commonly females and had chronic illnesses. MHA were more commonly younger, unemployed males who had longer referral delay than UM originating in other countries, but no significant statistical differences were found in the complexity of disease or the need for referral.

### Health care utilization

Most of the UM who visited both ambulatory walk-in clinics were young and single males, who stayed in Israel for a few months and complained of mild/simple and acute medical conditions. The most common work-related health problems reported among the migrants included respiratory and musculoskeletal involvement, in line with similar studies from developed countries [11].

MHA received special attention in our study due to the recent influx of migrants originating from those countries. These migrants were more likely to be younger single males than UM coming from other countries, reflecting the migrant communities residing in Israel [12, 13]. The majority were unemployed males who had longer referral delay from the start of the symptoms. This delay in referral may be associated with the shorter length of stay in Israel compared with migrants from other countries, who have been in Israel for longer periods and thus become more familiar with the availability of medical services. Additionally, MHA may lack family support, as the majority are single. Some may suffer from post-traumatic stress disorders due to harassments and attacks they experienced during their terrestrial voyage to Israel [14]. Interestingly, we did not find association between the country of origin and the severity of disease or the need for further referral to secondary and tertiary health care.

### Health services

The ambulatory clinics were established as a humanitarian aid in order to provide basic medical care to UM. The medical services in the clinics include physical examination as well as treatment, according to the expertise of the attending physicians or to the equipment available at the clinic. Although the nature of the symptoms in most cases is mild or simple, we have found that nearly 40% of the migrants necessitate a referral to another medical setting in order to complete the medical evaluation or to receive additional counselling/treatment. It seems that due to financial constraints of the clinics, the ambulatory services in most cases can mainly respond to acute medical problems, while preventive services for adults and long term care do not receive sufficient attention, as also found in migrant community clinics in other European countries [15]. Migrants who are referred to other medical settings may be confused by bureaucracy or become disorientated with the new environment. They usually try not to distant themselves from their comfort zone in south Tel-Aviv, as they are not safeguarded and may be identified by the migration police [16]. Consequently, referred migrants may avoid the recommended medical visits, while hampering the continuity of their medical care.

The complexity of the medical condition and proportion of referrals to other health settings in our study was not different between MHA and migrants from other countries. Yet, a greater proportion of women were referred to other medical settings, as a result of a greater need for gynecological care, which had not been available fully at the clinics. A recent publication from Israel has suggested that MHA have high rates of psychological problems as a result of stress and assault they experienced during their voyage to Israel [14]. It is therefore recommended that the clinics include gynecological and psychological secondary care consultation to meet the medical needs of the migrants.

Linguistic and cultural capabilities of the medical staff are key components in reducing existing barriers between the UM and health care providers [17]. In some cases, unsatisfactory communication may even result in improper medical decisions. Consequently, the clinics are making efforts to bridge those gaps by increasing the medical staffs’ awareness of the cultural diversity and employing community members as peers and translators to increase patient-providers’ confidence, improve communication and decrease health illiteracy among UM.

### Migrants’ health policy

UM are excluded from the National health insurance and are therefore not insured by their employers. Looking at the bigger picture, the lack of clear and consistent migration policy by the government of Israel, as in many receiving countries in the world, complicates future planning and precludes investing in human resource and infrastructure.

One possible solution to ameliorate the medico-social status of UM is to approve “social residence” and issue a work permit for the migrants, while obliging their employers to medically insure the UM. This solution is justified morally, considering health is a basic human right rather than a discretionary charity-based commodity, and it is also economically plausible [18]. Although some politicians have raised the concern that ensuring health insurance for the UM may encourage further undocumented migration waves, it has not been proven elsewhere [19]. Unfortunately, some politicians and policy makers expressed concerns that migrants can transmit infectious diseases and threaten the Israeli community. These ideas are somehow misleading, as most infections are transmitted between contacts who share households or work place, which is rather uncommon among the migrants and the wider Israeli community [20].

This study is subject to several methodological limitations. First, it included only migrants who visited the clinics, and therefore may not provide a complete assessment of the health status of the UM. Second, the diagnoses were not coded, but recorded as free text. We therefore classified the diagnoses according to the affected body system.

## Conclusions

UM who had mild or simple symptoms were mostly assisted by the ambulatory clinics. However, the health needs of women and migrants who had more complex medical conditions or chronic diseases were not commonly met, and they were referred to other medical settings, while jeopardizing the continuum of care. The newly equipped MoH clinic and the opening of the migrants’ mental health clinic opened in 2012 may provide a partial solution, yet a fresh and broader framework should be sought to ensure the social and medical well-being of UM.

## Abbreviations

MHA: Migrants from horn of Africa
MoH: Ministry of Health
PHR-I: Physicians for Human Rights- Israel
UM: Undocumented migrants
